# Active success drives momentum, but late-stage errors destroy it: a real-time analysis of volleyball

**DOI:** 10.3389/fpsyg.2026.1839311

**Published:** 2026-06-02

**Authors:** Yusuke Asai, Tomoyasu Okuda, Noriyuki Shide, Noriyuki Kida

**Affiliations:** 1 Department of Sport Cultural Studies, Hokkaido University of Education, Hokkaido, Japan; 2 Graduate School of Science and Technology, Kyoto Institute of Technology, Kyoto, Japan; 3 School of Humanities, Hokusei Gakuen University, Hokkaido, Japan; 4 Faculty of Arts and Sciences, Kyoto Institute of Technology, Kyoto, Japan

**Keywords:** attribution theory, crediting causality model, norm theory, overreliance, proxy dilemma

## Abstract

**Introduction:**

Psychological momentum is a dynamic system that characterizes the psychological states of individuals progressing toward or regressing from goal attainment. As research has largely relied on controlled laboratory settings or artificial scenarios, empirical evidence from actual athletic competitions remains limited. Therefore, this study aimed to comprehensively elucidate how play content, game stages, and player expertise interact to dictate the micro-dynamics of momentum among actors during a real match.

**Methods:**

Real-time measurements were conducted with 25 male high-school volleyball players during practice matches to ensure high ecological validity. Participants used voice recorders during natural intervals between rallies to verbally rate their momentum fluctuations. A dataset comprising 2,103 observations was analyzed using a linear mixed model to evaluate the fixed effects of expertise, game stage, play content (active success versus opponent error), and rally outcomes. Because the model did not include the lagged dependent variable, the first-order autoregressive [AR(1)] residual covariance structure was used only to address potential serial correlation in the residuals across adjacent rallies, rather than to model temporal carryover in psychological momentum itself.

**Results:**

Scoring through a team’s active success yielded significantly higher momentum improvements than scoring through an opponent’s error across all game stages. In the late stages of a set, conceding a point because of a self-induced error resulted in a significantly more severe deterioration of momentum than conceding a point through an opponent’s active success. Furthermore, advanced players exhibited a significantly greater decrease in momentum following lost points than intermediate players.

**Discussion:**

These findings suggest that momentum fluctuations are highly sensitive to players’ internal attributions of events, particularly the high mutability and regret associated with late-stage errors. Advanced players’ acute sensitivity to conceded points highlights the phenomenon of proxy agency and the risk of proxy dilemmas among less experienced teammates. Practically, coaches can mitigate this overreliance and enhance self-agency by assigning specific, specialized roles, such as defensive pillars or high-set providers, to every player, ensuring that all participants can exercise personal agency and earn mutual trust within the team.

## 1 Introduction

According to Iso-Ahola and Mobily (1980, p. 391–92), psychological momentum (PM) “is likely to increase the person’s self-confidence, thus improving his mental performance, i.e., attentiveness, concentration and thinking, and increasing the amount of mental and physical effort exerted.” An ongoing debate concerns the impact of PM on match outcomes. Some researchers argue that PM is a crucial determinant of success and failure in sports (Iso-Ahola and Mobily, 1980; Vallerand et al., 1988), whereas others contend that it is not (Cornelius et al., 1997). Nevertheless, PM continues to attract the interest of psychologists as it captures the psychological characteristics of individuals progressing toward or regressing from goal attainment (Gernigon et al., 2010, p. 377). PM is not a static construct but a dynamic system characterized by non-stationarity and history dependence (Gernigon et al., 2010). Therefore, to accurately understand PM, these continuously fluctuating psychological states must be captured in real time during the temporal flow of a match.

Most PM research has relied on virtual scenarios in laboratory settings or tasks with artificially manipulated scores (Perreault et al., 1998; Briki et al., 2013; Den Hartigh and Gernigon, 2018). Although a few studies have surveyed volleyball players during practice, they are limited to controlled experimental environments (Stanimirovic and Hanrahan, 2004). Actual athletic competitions, particularly open-skill ball games, involve uncontrollable chaotic elements, such as back-and-forth rallies and dramatic comebacks. Laboratory findings’ direct application to this complex “field” environment is not guaranteed, highlighting a potential lack of ecological validity. Furthermore, while many studies focus on “spectators,” “actors” on the court experience significantly more intense affective and motivational fluctuations than observers (Vallerand et al., 1988). Consequently, investigating athletes’ perception of PM during matches is critical for evidence-based coaching. Therefore, to ensure high ecological validity, this study investigated the PM of players during gameplay.

Furthermore, fluctuations in PM are triggered by “precipitating events,” and impressive plays often serve as catalysts (Vallerand et al., 1988; Iso-Ahola and Dotson, 2017). This impact should be particularly pronounced in net/wall games, in which the success or failure of a play directly translates into a point. Here, a critical unresolved question emerges: Does scoring through a team’s “active success” affect PM differently than scoring through an opponent’s error (“passive gain”)? Previous research has theorized that, owing to the zero-sum nature of sports, an opponent’s error also contributes to a team’s advantage (Iso-Ahola and Dotson, 2014). However, from a practical coaching perspective, whether a windfall point gained from an opponent’s unforced error generates the same momentum as a point earned through a powerful spike remains uncertain.

Although Wergin et al. (2019) explained collective team collapse in terms of a team’s own errors, this phenomenon represents a more distinctly negative state than PM. Given that Wergin et al. (2019) characterized PM as having a more fluid and variable nature compared to collective team collapse, there is clear value in elucidating PM through the lens of specific play content. Recently, Blything and Blything (2024) analyzed professional tennis matches by dichotomizing points into successes and errors to predict winning probabilities. However, because their analysis was based exclusively on objective game statistics, definitive claims about the direct relationship between PM and play content should be avoided, even though PM is discussed in their paper.

Moreover, in actual matches, PM fluctuations are not solely driven by the immediately preceding play. Beyond play content and scoring, PM perception is shaped by a complex interplay of various factors. For instance, Miller and Weinberg (1991) formulated a theoretical model positing that variables such as expertise level, changes in the match situation, game phases, and score differentials significantly influence PM; however, empirical evidence supporting these claims within real-world contexts remains scarce.

Therefore, PM cognition is a highly complex phenomenon involving numerous interacting variables. Categorizing these variables and elucidating their combined influence on PM will enable a deeper understanding of the psychological characteristics of athletes striving for goal attainment, thereby providing practical insights for bench staff coaching. This served as the primary research question of our study.

Studies suggest that these factors individually affect PM. However, it remains unclear how play content (active success vs. passive gain) and scoring interact with macro-level factors such as expertise and match outcomes to dictate the micro-dynamics of players’ PM within the complex context of an actual match. Therefore, to ensure high ecological validity, we conducted real-time measurements of “actors” during practice matches. This study aimed to comprehensively elucidate the multifaceted effects of expertise level, play content, scoring, changes in match situations, and game phases on PM cognition using a linear mixed model (LMM).

## 2 Methods

### 2.1 Participants

The participants were 25 male high-school volleyball players from three teams. Their ages ranged from 15 to 18 years (M = 16.31, SD = 0.93), and their competitive experience ranged from 1 to 11 years (M = 6.85, SD = 3.09). Regarding the competitive level, 10 players had experience competing in national tournaments in Japan, while 15 had no such experience. The study protocol was approved by the Ethics Committee of the Hokkaido University of Education (Approval no. 2025065003). After providing a written explanation of the study’s purpose and procedures, we obtained written informed consent from all participants and their legal guardians.

### 2.2 Procedures

Two practice matches were organized among the three teams. Team A played both matches, whereas Teams B and C played one match each. The matches were played in a best-of-three sets format, with 25 points required to win a set. Considering the composition of each team, coaches were asked to balance the competitive abilities of their squads to produce closely contested matches. Consistent with the aims of this study, the final rosters were required to include players with national tournament experience. To verify the match events, the entire duration of the matches was recorded using a video camera (FDR-AX45A; Sony).

### 2.3 Measures

- *Basic information:* Data on age, playing position, national tournament experience, and uniform numbers were collected using Google Forms.
- *Momentum:* Players wore a small IC voice recorder (MZ001; QZTELECTRONIC) attached to the inside of their nondominant upper arm. After each rally, they verbally recorded a numerical value indicating the extent to which their momentum had improved or worsened compared with the previous rally (e.g., +1 for slight improvement and −2 for deterioration). The range of values was unrestricted to avoid ceiling effects. While the ratings were not recorded at the start of the first set, players provided ratings at the beginning of the second set based on the outcome of the first set. To minimize interference with the game’s progression, ratings were recorded between rallies, and players were instructed to speak softly to ensure that the surrounding participants could not hear their ratings. Importantly, no additional time was allocated for these recordings. Instead, they were completed within the natural intervals between rallies to ensure ecological validity.
- *Experience:* The experience level was coded as 1 for players with Japanese national tournament experience and 2 for those without.
- *Game stage:* Given the confirmed importance of match situations (Miller and Weinberg, 1991), the sets were divided into three stages: early (until the leading team reached 8 points) = 1, middle (until the leading team reached 16 points) = 2, and late (until the end of the set) = 3. This division was based on the technical timeout scores in previous volleyball rules.
- *Play content:* The terminal action of each rally was evaluated by two raters (the first author and the coauthor). The criteria classified active plays leading to a point for the team (e.g., spikes, blocks, and serves) as “active success” and errors resulting in a lost point for the team (e.g., net touches, service errors, and ball-handling errors) as “error.” It is difficult to strictly distinguish between forced errors (e.g., reception or dig errors from powerful spikes) and unforced errors in real time; therefore, the operational definition treated any action that is not explicitly ruled a foul or error by the referee as the opponent’s “active success.” To ensure the reliability of the performance classification, both authors classified all plays independently. Both raters were volleyball experts with 12 and 40 years of coaching experience, respectively. Interrater reliability was assessed using Cohen’s kappa, based on the independent classification of 206 plays. The analysis revealed almost perfect agreement (*κ* = 0.96, *p* < 0.001). Minor discrepancies were resolved by consensus to finalize the dataset.
- *Outcome:* The outcomes of each rally were recorded. Scoring a point was coded as 1, and conceding a point was coded as 2.

### 2.4 Data analysis

- *Data preprocessing:* To eliminate individual bias related to rating scale usage tendencies (e.g., response amplitude and baselines), the cumulative sums of the rating values were intra-individually standardized (*Z*-scores). The difference (amount of change) between the consecutive time points of these standardized values was calculated and used as the dependent variable (momentum fluctuation value). This approach enabled the evaluation of relative momentum fluctuations within an individual rather than relying on absolute numerical magnitudes. When calculating this momentum fluctuation value (i.e., the difference between the momentum at Rallies 1 and 2), if a participant failed to record an entry for Rally 2, the difference between Rallies 1 and 3 could not be computed and was, therefore, treated as a missing value. The final dataset comprised 2,103 observations.
- *Statistical modeling:* A linear mixed model (LMM) was used to analyze the rally-level repeated-measures data. Let $Y_{\mathrm{igt}}$ denote the momentum change score for participant i , in game g , at rally t , and let $X_{\mathrm{igt}}$ denote the corresponding vector of explanatory variables. Estimation was based on the conditional distribution of $Y_{\mathrm{igt}}$ given $X_{\mathrm{igt}}.$ The final model was specified as,
$$Y_{\mathrm{igt}}=X_{\mathrm{igt}}^{\prime }\beta +b_{i}+\varepsilon_{\mathrm{igt}}$$
  Where $X_{\mathrm{igt}}$ included expertise, game stage, content, outcome, and interaction terms involving outcome that were retained on the basis of theoretical interpretability and estimation stability. The term $b_{i}$ represents a participant-specific random intercept introduced to account for the repeated-measures structure within participants, and was assumed to follow:
$$b_{i}\sim N(0,\tau^{2})$$
  To directly model temporal carryover in the observed momentum change score, a dynamic specification including the lagged dependent variable, $Y_{\mathrm{ig},t-1}$ , as a predictor would be required. We initially attempted to estimate this specification; however, due to the complexity of the model and data structure, it resulted in severe non-convergence issues in SPSS and did not yield stable estimates. Therefore, the final inferential model did not include the lagged dependent variable, and temporal carryover in the observed momentum change score was not directly tested in the present analysis. The first-order autoregressive [AR(1)] residual covariance structure was specified only to address potential serial correlation in the residuals across adjacent rallies within each participant-by-game series, rather than as a substitute for the lagged dependent variable model. Specifically, for the residual vector $\varepsilon_{\mathrm{ig}}={\left(\varepsilon_{\mathrm{ig}1},\ldots ,\varepsilon_{\mathrm{igT}}\right)}^{\prime }$ within participant i and game g ,
$$\varepsilon_{\mathrm{ig}}\sim N(0,\sigma^{2}R_{\mathrm{ig}}(\rho ))$$
  With,
$$\text{Corr}\;(\varepsilon_{\mathrm{igt}},\varepsilon_{\mathrm{igs}})=\rho^{|t-s|}$$
  Expertise, game stage, content, and outcome were treated as fixed effects because these effects themselves were the substantive targets of inference in the present study. By contrast, participant ID was treated as a random intercept not because individual differences were of direct substantive interest, but because it represented the clustering of repeated observations within participants. Team ID was initially considered as an additional random effect; however, because the number of teams was small and the corresponding variance component was estimated near the boundary, team-level random effects were not retained in the final model.
  Model assumptions were assessed through residual diagnostics. Visual inspection of Q-Q plots and residuals-versus-fitted plots indicated no substantial deviations from normality or homoscedasticity. By adopting an AR(1) residual covariance structure rather than an independence structure, the assumption of independence among residuals within each participant-by-game series was relaxed. However, the AR(1) residual parameter was not interpreted as evidence for, or against, temporal carryover in the observed momentum change score. When significant main effects or interactions were detected, *post hoc* multiple comparisons with Bonferroni adjustment were conducted. All analyses were performed using IBM SPSS Statistics 29.0, with the significance level set at α=0.05 .

## 3 Results

### 3.1 Model diagnostics and covariance structure

The final linear mixed model, which included a participant-specific random intercept and a first-order autoregressive [AR(1)] covariance structure for repeated observations within each participant-by-game series, converged successfully. Visual inspection of the Q-Q plot and the residuals-versus-fitted plot did not indicate substantial departures from normality or homoscedasticity. The estimated participant-level random intercept variance was very small (2.03 × 10^-4, *p* = 0.616), suggesting limited between-player heterogeneity in baseline momentum change scores. The estimated AR(1) residual correlation parameter was also small (rho = −0.051, *p* = 0.054), and did not provide clear evidence of substantial first-order residual serial correlation in the final specification. Nevertheless, the AR(1) residual covariance structure was retained to relax the independence assumption among residuals within each participant-by-game series. This AR(1) residual parameter was not interpreted as evidence for, or against, temporal carryover in the observed momentum change score.

Table 1 presents the *F* and *p* values for the main effects as well as the two- and three-way interactions derived from the LMM. Table 2 presents the estimated marginal means, standard errors, and 95% confidence intervals for each factor in the main effects. This study aimed to clarify the perceived direction (positive or negative) of momentum and the factors contributing to its fluctuation. The direction of PM was inherently determined by the play’s outcome (i.e., whether a point was won or lost). Therefore, the main effects and interactions that did not include the outcome (e.g., content × game stage) were excluded from the current discussion, as positive and negative PM values would cancel each other out, rendering interpretation difficult.

**Table 1:** Results of the linear mixed model for perceived momentum.

Source	Num *df*	Den *df*	*F*	*p*
Intercept	1	21.317	8.413	0.008
Experience	1	21.407	0.244	0.627
Game Stages	2	869.193	0.546	0.579
Content	1	2070.389	60.562	0.000
Outcome	1	2022.058	1855.248	0.000
Experience × Game Stages	2	869.552	0.497	0.608
Experience × Content	1	2068.477	0.981	0.322
Experience × Outcome	1	2037.252	10.597	0.001
Game Stages × Content	2	2063.673	1.345	0.261
Game Stages × Outcome	2	2026.744	1.392	0.249
Content × Outcome	1	1817.599	3.685	0.055
Experience × Game Stages × Content	2	2063.028	4.223	0.015
Experience × Game Stages × Outcome	2	1970.844	0.168	0.845
Experience × Content × Outcome	1	1862.683	0.422	0.516
Game Stages × Content × Outcome	2	2068.101	4.896	0.008

**Table 2:** Estimated marginal means and 95% confidence intervals for perceived momentum across main effects.

Factor	Level	EMM	(SE)	95% CI [LL, UL]
Experience	Advanced	0.019	(0.012)	−0.006 to 0.044
	Intermediate	0.026	(0.010)	0.006 to 0.047
Game Stages	Early	0.029	(0.013)	0.002 to 0.055
	Middle	0.026	(0.013)	0.000 to 0.052
	Late	0.012	(0.012)	−0.011 to 0.036
Content	Active Success	0.080	(0.009)	0.062 to 0.099
	Error	−0.035	(0.012)	−0.059 to −0.011
Outcome	Point Won	0.344	(0.011)	0.323 to 0.366
	Point Lost	−0.299	(0.011)	−0.321 to −0.278

**Figure 1:**
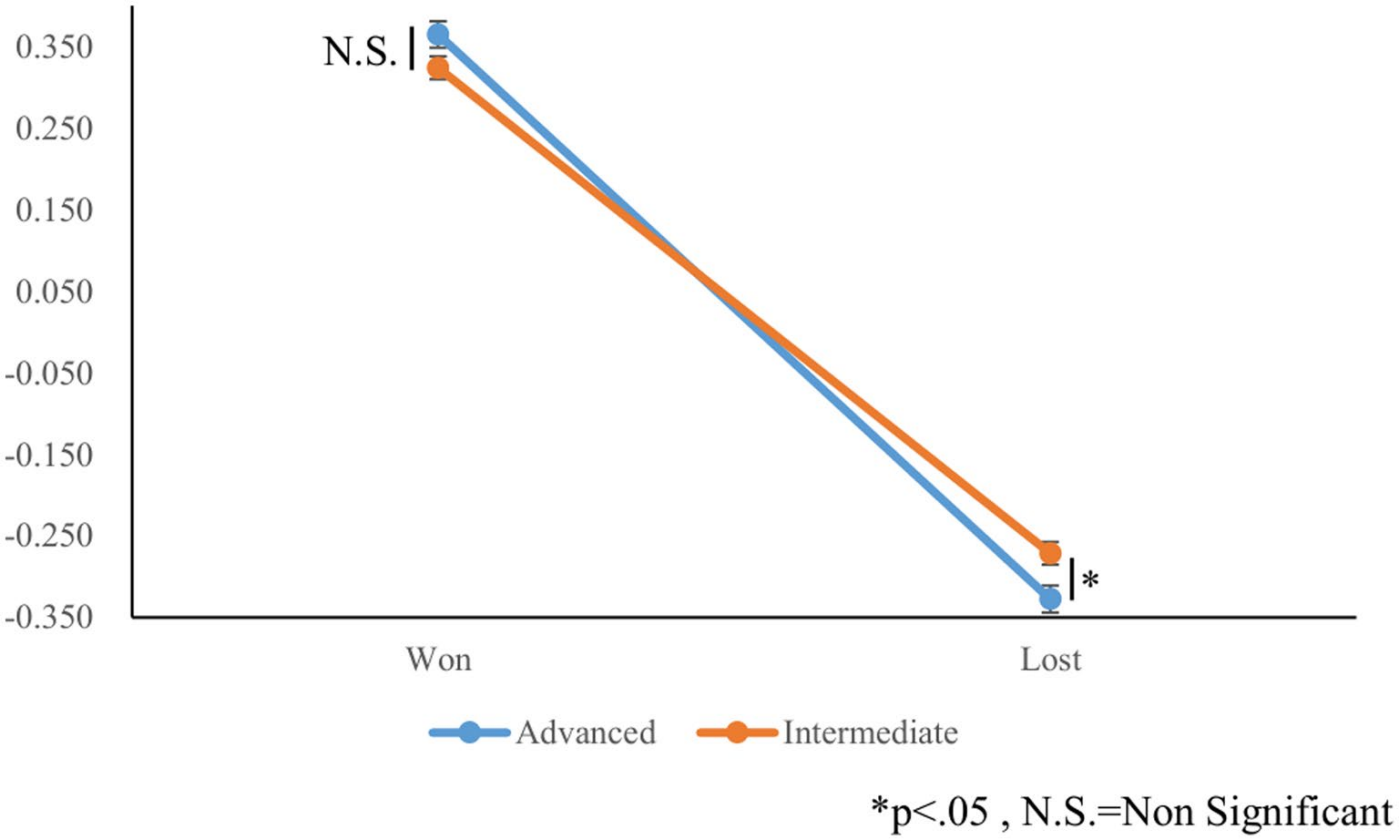
Interaction effects of expertise and rally outcome on psychological momentum.

**Figure 2:**
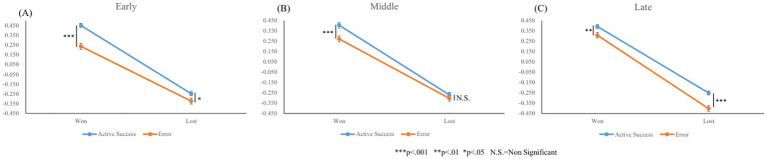
Three-way interaction effect of game stage, play content, and rally outcome on psychological momentum in the **(A)** Early, **(B)** Middle, and **(C)** Late game stages.

EMM, estimated marginal mean; SE, standard error; CI, confidence interval; LL, lower limit; UL, upper limit. Significant interactions are presented in Figures 1, 2.

### 3.2 Main effects

Significant main effects were observed for the content and outcome (Table 1). The specific values of the main effects are listed in Table 2. Regarding the content, active success yielded significantly higher PM values than errors. For the outcome, winning yielded significantly higher values than losing.

### 3.3 Two-way interactions

Significant two-way interactions were found only for experience × outcome, *F*(1, 2,037.25) = 10.60, *p* < 0.001. *Post-hoc* analyses of the experience × outcome interaction (Figure 1) revealed no significant difference between experience levels when a point was won (*p* = 0.060). However, when a point was lost, players with national tournament experience exhibited significantly lower PM (*p* = 0.011).

### 3.4 Three-way interactions

A significant three-way interaction was observed for experience × game stage × content, *F*(2, 2,063.03) = 4.22, *p* = 0.015. However, because this interaction did not include the outcome, it could not be interpreted in terms of PM direction and was, thus, excluded from the discussion. Another significant three-way interaction was found for game stage × content × outcome, *F*(2, 2,068.10) = 4.90, *p* = 0.008. *Post-hoc* tests (Figure 2) revealed that PM values were significantly higher when active success resulted in a won point across all game stages: early (*p* < 0.001), middle (*p* < 0.001), and late (*p* = 0.008). Furthermore, for lost points in the early and late stages, errors resulted in significantly lower PM values (early: *p* = 0.047, late: *p* < 0.001).

## 4 Discussion

The primary finding of this study is that momentum fluctuations in volleyball, triggered by scoring or conceding points, were dictated by a complex interaction between game stages, play content, and player expertise. Specifically, the results highlighted the uniquely detrimental impact of self-induced errors on PM during the late stages of a set and the sensitive reactions of advanced players to conceded points. Based on the results of the LMM and considering the hierarchy and interpretability of interactions, this discussion focuses on two major findings: (1) the shifting significance of points won or lost across game stages (game stage × content × outcome) and (2) the differences in how conceded points are evaluated based on expertise level (experience × outcome). To maintain a logical narrative flow, we first discuss the results in Figure 2, followed by those in Figure 1.

### 4.1 Shifting significance of points won or lost across game stages (game stage × content × outcome)

Regarding the game stage × content × outcome interaction, scoring a point (won) through active success yielded significantly greater PM improvements than scoring through the opponent’s error across all stages. This pattern is consistent with attribution theory (Weiner, 1985), in that points gained through one’s own successful play may be perceived as more self-generated and controllable than points gained through the opponent’s mistake. As a result, momentum improvement following active success may be stronger than that following the opponent’s error. By contrast, for conceded points (lost), the magnitude of PM deterioration showed a stage-dependent pattern. A significant difference between points lost through the opponent’s active success and those lost through one’s own error was absent only in the middle stage, but emerged in both the early and late stages, with the largest decline observed in the late stage (Figure 2). This pattern may be interpreted in light of norm theory (Kahneman and Miller, 1986), which suggests that errors are highly mutable events because they are easily subjected to counterfactual thinking (e.g., “I should have done this differently”). Such mutability may amplify negative emotional responses, including regret, when a point is lost through one’s own error rather than through the opponent’s good play. One possible interpretation is that, during the middle stage, players may perceive greater opportunity for recovery, thereby attenuating the differential impact of play content on PM following lost rallies. In contrast, own errors may be especially disruptive in the early stage, when teams are still establishing rhythm, and particularly damaging in the late stage, when each rally carries greater consequence. Furthermore, the credit causality model (Spellman, 1997) may help explain why the decline in PM appeared largest in the late stage (Figure 2C). This framework proposes that greater responsibility is attributed to events perceived to have stronger consequences for the final outcome. Thus, an error occurring in the decisive late stage may elicit heightened regret and perceived responsibility, hindering momentum beyond the mere loss of a single point.

Furthermore, one of the most compelling findings of the current study is that while the two-way interaction between Content and Outcome was not statistically significant on its own, the three-way interaction including match Stage (Stage × Content × Outcome) emerged as highly significant. This highlights the highly contextual and dynamic nature of psychological momentum (PM). It demonstrates that the psychological impact of a specific play and its result is not universal; rather, it is fundamentally dependent on when it occurs during the match. A combination of play content and outcome that might have a negligible effect on PM during the early stages of a match can become a critical trigger for momentum shifts during the late, high-stakes phases. By empirically capturing this phase-dependent volatility, our study extends the current literature, emphasizing that PM cannot be fully understood without accounting for the temporal context of the match.

Visually, the interaction patterns depicted in Panel C (Late stage) diverge markedly from those in Panels A and B (Early and Middle stages). Specifically, the trajectory of Psychological Momentum (PM) demonstrates a severe, precipitous decline when points are lost due to a team’s own errors in the final stage—a sensitivity that is far less pronounced earlier in the match. Although Den Hartigh et al. (2020) indicated in their study on psychological momentum (PM) in soccer that conceding a goal in the late stage of a match deteriorates PM more severely than in the middle stage, our analytical results did not reveal a significant difference in the impact of simply losing a point across the early, middle, and late stages (Game Stages × Outcome: *F*(2, 2026.74) = 1.39, *p* = 0.249). Therefore, strictly comparing the two studies reveals a discrepancy in the findings. Rather, the primary assertion of the current study is that the content generating the outcome, and specifically *when* it occurs during the match, is what makes the critical difference in PM.

### 4.2 Differences in the evaluation of conceded points based on expertise (experience × outcome)

As the sample size for this analysis was 25, the generalizability of the between-person effects based on individual attributes was constrained. Consequently, the estimates in this section may entail uncertainty (i.e., wider confidence intervals), and the results should be interpreted with caution. Some of these findings are exploratory and require further replication and verification using data from a more diverse range of teams and matches. Regarding the experience × outcome interaction, advanced players exhibited a significantly greater decrease in momentum following a lost point than intermediate players (Figure 1). This indicates that advanced players understand the “pain” of conceding a point more acutely than their less experienced counterparts. Previous research suggests that advanced players excel at tactical decision-making and situational awareness when trailing or after losing a point (Fruchart et al., 2010). Given this, our results implied that advanced players perceive a single conceded point as a significant threat that cannot be overlooked and that this acute awareness shapes their foresight and anticipation of the match’s progression. However, intermediate players are likely to lack this foresight regarding shifting match dynamics. This difference can be explained by the concept of proxy agency, with intermediate players being likely to rely on advanced players’ capabilities. Proxy agency occurs when individuals try to achieve desired outcomes by relying on a competent proxy to act on their behalf in situations they cannot control (Bandura, 2006; Alavi and McCormick, 2016). This choice stems from an understanding of their lack of ability. Relying on advanced players represents an appropriate utilization of resources toward the goals of scoring and winning. However, overreliance on a proxy can lead to negative consequences, that is, the “proxy dilemma” (Bandura, 2006), and our study might have captured a glimpse of this phenomenon. Although proxy agency can positively affect self-efficacy when used appropriately, overreliance has detrimental effects. In other words, our findings suggest that by relying too heavily on others, intermediate players stopped exercising their “personal agency.” Consequently, unlike advanced players, intermediate players did not perceive a severe deterioration in momentum after a lost point. Conversely, after a winning point, they recognized the contributions of the advanced players rather than attributing the success to their own efforts, leading to a more moderate improvement in momentum compared with advanced players. Data that clearly demonstrate such differences in momentum perception between advanced and intermediate players are rare. While a relationship between winning/losing and momentum has long been suggested (Iso-Ahola and Mobily, 1980), it is often unclear whether the outcomes are the result of actual ability or the influence of momentum. This makes the relationship between performance and momentum appear ambiguous (Briki, 2017). However, the present study revealed a clear disparity in how advanced and intermediate volleyball players grasp momentum as a form of situational cognition. Previous studies comparing competitive levels have often relied on the simple dichotomy of having or lacking experience in the sport (Vallerand et al., 1988). This study demonstrated that highly skilled players perceive momentum differently than less skilled players; this finding will likely accelerate future research aimed at clarifying the complex relationship between performance and PM.

### 4.3 Implications for coaching

Based on the insights of this study, it would be highly effective to assign specific roles to every player within a rally rather than categorizing players within the rigid framework of “advanced” versus “intermediate.” This will encourage each athlete to exercise their personal agency while allowing players to mutually exercise proxy agency toward one another’s distinct roles. In other words, by developing team members into specialists who score points by fulfilling their specific duties, the distinction between advanced and intermediate players dissolves; instead, it becomes crucial for all participants to perform as “advanced players” in their respective roles. For instance, a player does not always need to score; serving as a defensive pillar as a receiver specialist or consistently delivering high sets that allow spikers to attack difficult balls is more than sufficient. Importantly, the player earns the trust of their teammates through the dedicated fulfillment of that specific role. This approach prevents overreliance on a single proxy and enables proxy agency to function effectively in a mutually respectful manner, thereby enhancing collective self-efficacy. While the proxy dilemma is less likely to occur when members’ roles are clearly defined, the disparity in participants’ abilities in this study underscores a critical need. To avoid overreliance on specific players, coaches must consciously clarify roles, encourage active effort in fulfilling these roles, and explicitly define the standards for role achievement.

### 4.4 Limitations

As this study prioritized ensuring ecological validity in a real-world setting, the generalizability of these findings is limited to volleyball. Because the perception of PM is highly dependent on the unique rules and scoring systems of a given sport, conducting experiments with high ecological validity across other sports is necessary to broaden the scope of these findings. Furthermore, players immersed in the intensity of the game occasionally forgot to record their data. However, deliberately allocating time or verbally prompting participants to record data between rallies could compromise the ecological validity the study sought to achieve; thus, a careful methodological balance is required for future field research. Additionally, although we successfully captured real-time PM, the reliance on a single-item subjective self-report leaves room for potential reporting bias. Future studies should employ multimodal approaches, combining self-reports with physiological data or objective performance metrics, to capture momentum more comprehensively. Finally, regarding the statistical analysis, the interpretation of the between-person effect (i.e., expertise level) requires caution. While the within-person effect was analyzed using a robust dataset of 2,103 observations, the between-person effect was based on a sample of only 25 participants from just three teams. This relatively small sample size at the upper levels not only limits generalizability but also precludes the statistical examination of team-level random effects (e.g., team culture or specific tactical instructions). Further replication with a larger and more diverse sample of teams is necessary to validate these between-person differences. Furthermore, from an analytical perspective, the residual AR(1) covariance structure was used only to address potential serial correlation in the residuals and to reduce potential standard error bias; it was not used to model temporal carryover in the observed momentum change score itself. To directly test temporal carryover in the observed momentum change score, a dynamic specification including the lagged dependent variable, $Y_{\mathrm{ig},t-1}$ , as a predictor would have been necessary. Although we attempted to estimate such a model, it did not converge and failed to yield stable estimates. Therefore, the final model did not include the lagged dependent variable, and the present study could not directly examine whether previous momentum change predicted subsequent momentum change. Future research should examine such temporal carryover processes using larger datasets and statistical approaches specifically suited to dynamic repeated-measures models.

## 5 Conclusion

This study elucidated the mechanisms underlying momentum fluctuations in volleyball matches by applying an LMM to real-world game data. The team’s active success consistently improved PM across all game stages. Furthermore, points conceded due to errors severely deteriorated PM during the late stages of a match. This suggests that the perceived inability to control one’s own play in critical situations directly linked to match outcomes exacerbates the decline in PM. A crucial implication is that by assigning specific roles to each player and clearly defining the standards for fulfilling these roles, coaches can enable athletes to maximize their performance without being hindered by conscious disparities in skill levels. However, the generalizability of these results is limited because the sample consisted solely of male high-school volleyball players. Missing data entries—a challenge inherent to field experiments—constitute another limitation that should be addressed in future research.

## Data Availability

The raw data supporting the conclusions of this article will be made available by the authors, without undue reservation.

## References

[ref1] Alavi S. B. McCormick J. (2016). Implications of proxy efficacy for studies of team leadership in organizational settings. Eur. Psychol. 21, 218–228. doi: 10.1027/1016-9040/a000270

[ref2] Bandura A. (2006). Toward a psychology of human agency. Perspect. Psychol. Sci. 1, 164–180. doi: 10.1111/j.1745-6916.2006.00011.x, 26151469

[ref3] Blything L. P. Blything R. (2024). The psychological (ab)use of timeouts in professional tennis. Int. J. Perform. Anal. Sport 26:2436271. doi: 10.1080/24748668.2024.2436271

[ref4] Briki W. (2017). Rethinking the relationship between momentum and sport performance: toward an integrative perspective. Psychol. Sport Exerc. 30, 38–44. doi: 10.1016/j.psychsport.2017.02.002

[ref5] Briki W. Den Hartigh R. J. R. Markman K. D. Micallef J. P. Gernigon C. (2013). How psychological momentum changes in athletes during a sport competition. Psychol. Sport Exerc. 14, 389–396. doi: 10.1016/j.psychsport.2012.11.009

[ref6] Cornelius A. Silva J. M. III Conroy D. E. Petersen G. (1997). The projected performance model: relating cognitive and performance antecedents of psychological momentum. Percept. Mot. Skills 84, 475–485. doi: 10.2466/pms.1997.84.2.475

[ref7] Den Hartigh R. J. R. Gernigon C. (2018). Time-out! How psychological momentum builds up and breaks down in table tennis. J. Sports Sci. 36, 2732–2737. doi: 10.1080/02640414.2018.1477419, 29785873

[ref8] Den Hartigh R. J. R. Van Yperen N. W. Gernigon C. (2020). Psychological momentum in football: the impact of a last-minute equalizer in a knock-out match. Sci. Med. Footb. 4, 178–181. doi: 10.1080/24733938.2019.1665704

[ref9] Fruchart E. Pâques P. Mullet E. (2010). Decision-making in basketball and handball games: a developmental perspective. Eur. Rev. Appl. Psychol. 60, 27–34. doi: 10.1016/j.erap.2009.10.003

[ref10] Gernigon C. Briki W. Eykens K. (2010). The dynamics of psychological momentum in sport: the role of ongoing history of performance patterns. J. Sport Exerc. Psychol. 32, 377–400. doi: 10.1123/jsep.32.3.377, 20587824

[ref11] Iso-Ahola S. E. Dotson C. O. (2014). Psychological momentum: why success breeds success. Rev. Gen. Psychol. 18, 19–33. doi: 10.1037/a0036406

[ref12] Iso-Ahola S. E. Dotson C. O. (2017). Momentum and elite performance. Nat. Sci. 3:e345.

[ref13] Iso-Ahola S. E. Mobily K. (1980). ‘Psychological momentum’: a phenomenon and an empirical (unobtrusive) validation of its influence in a competitive sport tournament. Psychol. Rep. 46, 391–401. doi: 10.2466/pr0.1980.46.2.391

[ref14] Kahneman D. Miller D. T. (1986). Norm theory: comparing reality to its alternatives. Psychol. Rev. 93, 136–153. doi: 10.1037/0033-295X.93.2.136

[ref15] Miller S. Weinberg R. (1991). Perceptions of psychological momentum and their relationship to performance. Sport Psychol. 5, 211–222. doi: 10.1123/tsp.5.3.211

[ref16] Perreault S. Vallerand R. J. Montgomery D. Provencher P. (1998). Coming from behind: on the effect of psychological momentum on sport performance. J. Sport Exerc. Psychol. 20, 421–436. doi: 10.1123/jsep.20.4.421

[ref17] Spellman B. A. (1997). Crediting causality. J. Exp. Psychol. Gen. 126, 323–348. doi: 10.1037/0096-3445.126.4.323

[ref18] Stanimirovic R. Hanrahan S. J. (2004). Efficacy, affect, and teams: is momentum a misnomer? Int. J. Sport Exerc. Psychol. 2, 43–62. doi: 10.1080/1612197X.2004.9671732

[ref19] Vallerand R. J. Colavecchio P. G. Pelletier L. G. (1988). Psychological momentum and performance inferences: a preliminary test of the antecedents-consequences psychological momentum model. J. Sport Exerc. Psychol. 10, 92–108. doi: 10.1123/jsep.10.1.92

[ref20] Weiner B. (1985). An attributional theory of achievement motivation and emotion. Psychol. Rev. 92, 548–573. doi: 10.1037/0033-295X.92.4.548, 3903815

[ref21] Wergin V. V. Mallett C. J. Mesagno C. Zimanyi Z. Beckmann J. (2019). When you watch your team fall apart – coaches' and sport psychologists' perceptions on causes of collective sport team collapse. Front. Psychol. 10:1331. doi: 10.3389/fpsyg.2019.01331, 31244730 PMC6581729

