# Supplementary material for: Active success drives momentum, but late-stage errors destroy it: a real-time analysis of volleyball
Source: Front Psychol. 2026 Jun 2;17:1839311. doi: 10.3389/fpsyg.2026.1839311 (PMC13269402; doi:10.3389/fpsyg.2026.1839311)
Supplement: Supplementary file 1 [file Data_Sheet_1.PDF]

Supplementary Material

Details of the Non-Convergent Lagged Dependent Variable Model

Manuscript title: Active Success Drives Momentum, But Late-Stage Errors Destroy It:

A Real-Time Analysis of Volleyball

Purpose

This supplementary material provides additional information on the lagged dependent variable model that was attempted during the revision process. The purpose of this model was to examine whether the immediately preceding psychological momentum score predicted the current psychological momentum score, while retaining the fixed-effect structure used in the main analysis. However, the model did not achieve convergence in SPSS, and therefore its parameter estimates were not interpreted or used for statistical inference in the manuscript.

Attempted model specification

The attempted linear mixed model used PM as the dependent variable and included Lag1_PM, defined as the psychological momentum score from the immediately preceding rally, as a fixed-effect predictor. The fixed effects included expertise level, game stage, play content, outcome, Lag1_PM, and the interaction terms retained in the main analysis. A participant-level random intercept was specified to account for repeated observations nested within players. The model was estimated using restricted maximum likelihood (REML), with Satterthwaite degrees of freedom.

Model specification for the attempted model

The model specification can be summarized as follows:

```
MIXED PM BY Expertise Game_stage Content Outcome WITH Lag1_PM
  /CRITERIA=DFMETHOD(SATTERTHWAITE) CIN(95) MXITER(500) MXSTEP(10) SCORING(1)
    SINGULAR(0.000000000001) HCONVERGE(0.00000001, RELATIVE)
    LCONVERGE(0, ABSOLUTE) PCONVERGE(0, ABSOLUTE)
  /FIXED=Expertise Game_stage Content Outcome Lag1_PM
    Expertise*Game_stage Expertise*Content Expertise*Outcome
    Game_stage*Content Game_stage*Outcome Content*Outcome
    Expertise*Game_stage*Content Expertise*Game_stage*Outcome
    Expertise*Content*Outcome Game_stage*Content*Outcome | SSTYPE(3)
  /METHOD=REML
  /RANDOM=INTERCEPT | SUBJECT(ID) COVTYPE(VC).
```

Convergence diagnostic output

SPSS issued the following warning for this model:

"Iterations terminated, but convergence was not achieved. The MIXED procedure ignores this warning. The subsequent results are based on the last iteration. The validity of the model fit is uncertain."

Treatment of the non-convergent output

Although SPSS produced output after the warning, the estimates were based on the final iteration of a model that did not converge. Because the validity of the model fit was uncertain, these estimates were not interpreted and were not used to draw conclusions in the manuscript. Accordingly, the present study does not directly test temporal carryover in psychological momentum through a lagged dependent variable model. This issue is acknowledged as a limitation, and future research should examine temporal carryover using statistical approaches that can provide stable estimation for lagged psychological momentum models.

Summary

In summary, a lagged dependent variable model including Lag1_PM was attempted, but SPSS reported non-convergence. Consequently, the model was not used for inference. The AR(1) residual covariance structure retained in the final analysis should therefore be interpreted only as a method for addressing potential serial correlation in the residuals and reducing possible standard error bias, not as a direct model of temporal carryover in psychological momentum itself.
