# Supplementary figures and images for: Active success drives momentum, but late-stage errors destroy it: a real-time analysis of volleyball
Source: Front Psychol. 2026 Jun 2;17:1839311. doi: 10.3389/fpsyg.2026.1839311 (PMC13269402; doi:10.3389/fpsyg.2026.1839311)

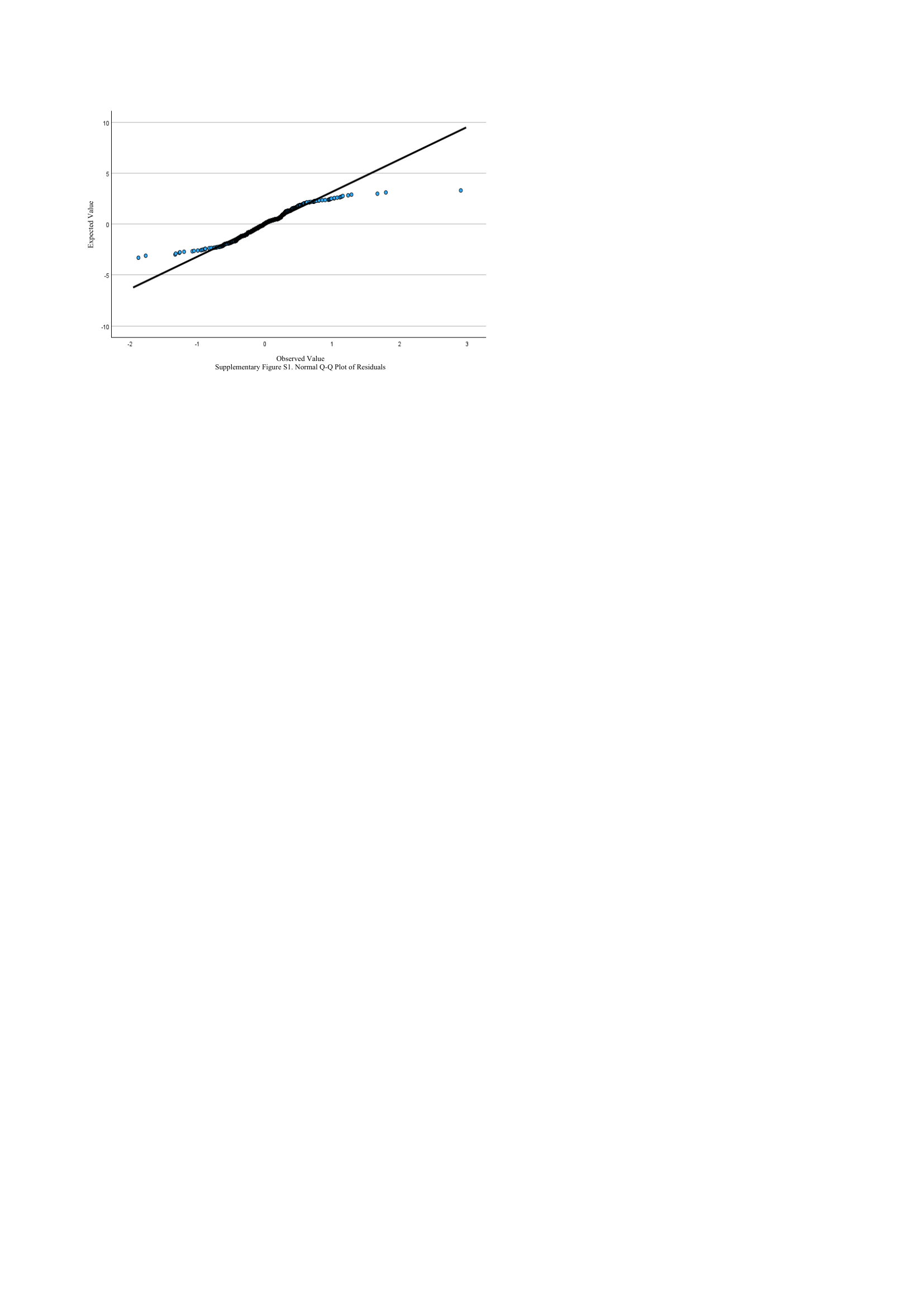

Supplement: Supplementary file 2 [file Image_1.TIFF]

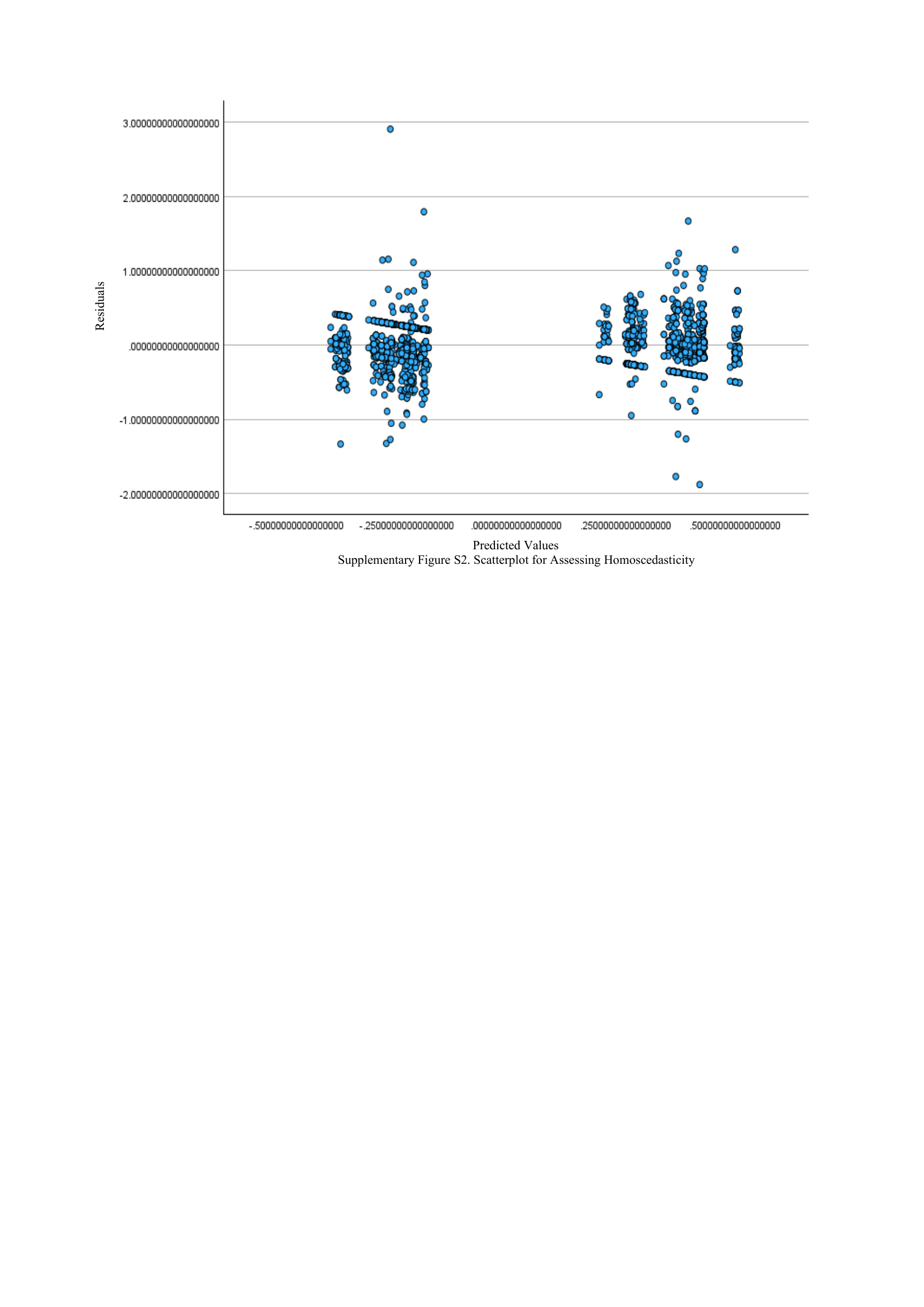

Supplement: Supplementary file 3 [file Image_2.TIFF]
